# Army Medical Coprs Examination

**Published:** 1916-11

**Authors:** 


### Army Medical Coprs Examination.

The surgeon general of the army announces that preliminary examination for appointment of first lieutenants in the Army Medical Corps will be held early in January, 1917, at points to be hereafter designated.

Full information concerning this examination can be procured upon application to the "Surgeon General, U. S. Army, Washington, D. C." The essential requirements to secure an invitation are that the applicant shall be a citizen of the United States, between 22 and 32 years of age at time of receiving commission in the Medical Corps, a graduate of a medical school legally authorized to confer the degree of Doctor of Medicine, of good moral character and habits, and shall have had at least one year's hospital training as an interne, after graduation. Applicants who are serving this post-graduate interneship and can complete same before October 1, 1917, can take the January examination. The examination will be held simultaneously throughout the country at points where boards can be convened. Due consideration will be given to localities from which applications are received, in order to lessen the traveling expenses of applicants as much as possible.

In order to perfect all necessary arrangements for the examination, applications should be forwarded without delay to the surgeon general of the army.

There are at present 228 vacancies in the Medical Corps of the Army.

